# Asthma Exacerbation Prediction and Risk Factor Analysis Based on a Time-Sensitive, Attentive Neural Network: Retrospective Cohort Study

**DOI:** 10.2196/16981

**Published:** 2020-07-31

**Authors:** Yang Xiang, Hangyu Ji, Yujia Zhou, Fang Li, Jingcheng Du, Laila Rasmy, Stephen Wu, W Jim Zheng, Hua Xu, Degui Zhi, Yaoyun Zhang, Cui Tao

**Affiliations:** 1 School of Biomedical Informatics The University of Texas Health Science Center at Houston Houston, TX United States; 2 Division of Gastroenterology Guang'anmen Hospital China Academy of Chinese Medical Sciences Beijing China

**Keywords:** asthma, deep learning, electronic health records, health risk appraisal

## Abstract

**Background:**

Asthma exacerbation is an acute or subacute episode of progressive worsening of asthma symptoms and can have a significant impact on patients’ quality of life. However, efficient methods that can help *identify personalized risk factors and make early predictions* are lacking.

**Objective:**

This study aims to use advanced deep learning models to better predict the risk of asthma exacerbations and to explore potential risk factors involved in progressive asthma.

**Methods:**

We proposed a novel time-sensitive, attentive neural network to predict asthma exacerbation using clinical variables from large electronic health records. The clinical variables were collected from the Cerner Health Facts database between 1992 and 2015, including 31,433 adult patients with asthma. Interpretations on both patient and cohort levels were investigated based on the model parameters.

**Results:**

The proposed model obtained an area under the curve value of 0.7003 through a five-fold cross-validation, which outperformed the baseline methods. The results also demonstrated that the addition of elapsed time embeddings considerably improved the prediction performance. Further analysis observed diverse distributions of contributing factors across patients as well as some possible cohort-level risk factors, which could be found supporting evidence from peer-reviewed literature such as respiratory diseases and esophageal reflux.

**Conclusions:**

The proposed neural network model performed better than previous methods for the prediction of asthma exacerbation. We believe that personalized risk scores and analyses of contributing factors can help clinicians better assess the individual’s level of disease progression and afford the opportunity to adjust treatment, prevent exacerbation, and improve outcomes.

## Introduction

### Background

Asthma is a common and serious health problem that affects 235 million people worldwide [1] and an estimated 26.5 million people (8.3% of the US population) in the United States [2]. Asthma takes a significant toll on the population, which imposes an unacceptable burden on health care systems. In 2013, the total annual cost of asthma was US $81.9 billion in the United States [3]. If not well controlled or stimulated by specific risk factors, asthma may develop into exacerbations (asthma attacks), which are acute or subacute episodes characterized by a progressive increase in one or more typical symptoms of asthma (dyspnea, coughing, wheezing, and chest tightness) [4]. In 2016, 12.4 million current asthmatics (46.9%) in the United States had at least one asthma exacerbation in the previous year [2]. Exacerbations of asthma can be severe and require immediate medical interventions, either as an emergency department (ED) visit or admission to the hospital [5]. Serious asthma exacerbations may even result in death [6]. Therefore, it is of practical significance to make early predictions such that interventions can be carried out in advance to reduce the probability of an exacerbation.

Investigations on risk factor analysis or prediction for asthma exacerbation have been respectable, in which the mainstream adopts traditional statistical methods, such as logistic regression [7-10], proportional hazards regression [11], and generalized linear mixed models [12]. However, most of them have only explored a small group of candidate risk factors and are usually hard to extend to other data sets and make personalized predictions difficult [13,14]. With the explosion of health care data in recent years, machine learning methods have taken a nontrivial place in this domain, benefiting from their general superiority over statistical methods in processing larger numbers of variables and flexibility in modeling more complex correlations [15]. Typical models include naïve Bayes [16], Bayesian networks [16-19], artificial neural networks [17], Gaussian process [17], and support vector machines [16,17]. However, although different attempts have been made, there are still several deficiencies in traditional machine learning methods. For example, ignoring temporal dependencies between variables might not provide a meaningful risk estimation of future exacerbations for individual patients [14]. Furthermore, most approaches only concentrate on the quantitative evaluation of prediction performance, but lack further attention to personalized risk factors [20].

Recent revolutions in health artificial intelligence started from deep learning, which has an upper hand on health care predictions because of its flexibility in dealing with longitudinal data [21], powerful learning capabilities [22], and ability to alleviate the problem of data irregularity [23]. One of the most popular architectures is recurrent neural networks (RNNs), which make predictions according to the sequence of historical events. Dozens of successes have been achieved in applying deep learning to disease predictions [24], mostly using variants of RNNs with distinct network components, for example, by adding an attention mechanism to evaluate the weights of each variable [25-29] or by using special configurations to tackle the problem of time decays [23,25,27,30-32]. Typical prediction tasks include the prediction of diabetes mellitus [23], Parkinson disease [29,33], chronic heart failure [26], sepsis [34], mortality, and readmission [25]. However, deep learning–based studies on the prediction of asthma exacerbation remain lacking. Do et al [35] proposed a protocol for the prediction based on RNNs and reinforcement learning but did not test the method on real-world data.

### Objectives

Inspired by previous studies, we applied long short-term memory (LSTM) [33], a popular RNN variant commonly used by previous predictive models [23-25,29,34] as the main framework for asthma exacerbation prediction, which can mitigate the gradient vanishing problem in RNNs. We proposed the time-sensitive, attentive neural network (TSANN), which employs a self-attention mechanism [36] to help model the context of both visit-level and code-level variables. Meanwhile, to incorporate the impact of elapsed time, we projected the relative time of each clinical variable into a low-dimensional space and combined it with the code representations. Using the attention weights of the TSANN, data analysis was then conducted to investigate personalized and cohort-level risk factors.

There are major differences between TSANN and recent state-of-the-art deep learning–based clinical predictive models such as time-aware LSTM (TLSTM) [23], Reverse Time Attention model (RETAIN) [27], and Attention-based Time-aware Disease Progression (ATTAIN) [32]. First, the model structures are different. Compared with TLSTM and ATTAIN, which only include 1 layer of RNN, our two-layer architecture enables us to analyze the relative importance of each event within each visit. Although RETAIN also has 2 layers of attention, it does not have explicit hierarchical structures as TSANN. Instead, an additional inference step is required to obtain the contribution of each variable. Second, TSANN uses a different approach to model the elapsed time. RETAIN, TLSTM, and ATTAIN feed the time elapsed into a decay function as a single value and multiply it with the network memory. In comparison, the elapsed time embeddings in TSANN are more analogous to position embeddings in natural language processing, which were introduced to model the relative distance between words by learning multidimensional and semantic representations to facilitate certain tasks such as relation classification [37] and neural language modeling [36,38,39]. By using time embeddings, we assume that time is no longer a single value as it was used in previous methods, but it can represent more complex patterns together with clinical variables such as varying lengths of correlations between variables.

The primary aims of this study were (1) to propose a novel predictive model with better performance and (2) to add the transparency of the model by visualizing contributing factors at both the individual and cohort levels. Furthermore, the proposed model can potentially be applied to other clinical problems. Deep learning models are usually scalable. Although focusing on asthma exacerbation for this specific project, the proposed approach can also be adopted in risk predictions for other chronic diseases. We hope that the associated pipeline of deep learning–based predictive modeling, including data collection, model training, model evaluation, and risk factor analysis, can help the clinical community better understand the underlying mechanisms of disease progression and assist in decision making.

## Methods

### Problem Statement

Given a sequence of historical clinical variables in patients with asthma, we aimed to evaluate the risk of developing asthma exacerbation in the designated time window. Meanwhile, personalized contributing factors are to be identified to facilitate the evaluation of disease progression and make early interventions.

### Database

The study used Cerner Health Facts, a Health Insurance Portability and Accountability Act–compliant database collected from multiple enrolled clinical facilities, containing mostly inpatient data. Data in Health Facts were extracted directly from the electronic health records (EHRs) from hospitals with which Cerner has a data use agreement. Encounters may include the pharmacy, clinical and microbiology laboratory, admission, and billing information from affiliated patient care locations. All personal identifying information of the patients was anonymized. In this study, we primarily focused on the impact of clinical factors on asthma exacerbation; therefore, we extracted diagnoses, medications, and demographic characteristics such as gender, race, and age from the database as clinical variables or clinical events. The University of Texas Health Science Center (UTHealth) had agreements with Cerner to use these data for research purposes. The institutional review board at the UTHealth approved the study protocol.

### Study Design

We conducted a retrospective study to predict the risk of asthma exacerbation. Patients’ records between 1992 and 2015 were extracted from the Cerner database. For clarity, we defined several terms in advance (Table 1).

We built 2 cohorts as simulations for both the real-world application scenario (*early prediction*) and the model evaluation (called *next-visit prediction* in many previous studies [27,32]). In early prediction, we could not foresee when the exacerbation would happen but could only evaluate the future risk at each visit. In our study, we selected the fifth visit from the asthma index as the prediction date (*testing set A*) according to the average number of visits (5.78, SD 6.04) between asthma index and exacerbation among the patients. The detailed steps for the cohort selection are listed in Multimedia Appendix 1 [4,25,40-55]. In next-visit prediction, we simply set the penultimate visit as the prediction date (*testing set B*). We set testing set A as our primary evaluation set as it was much closer to the realistic diagnostic situation.

**Figure 1:**
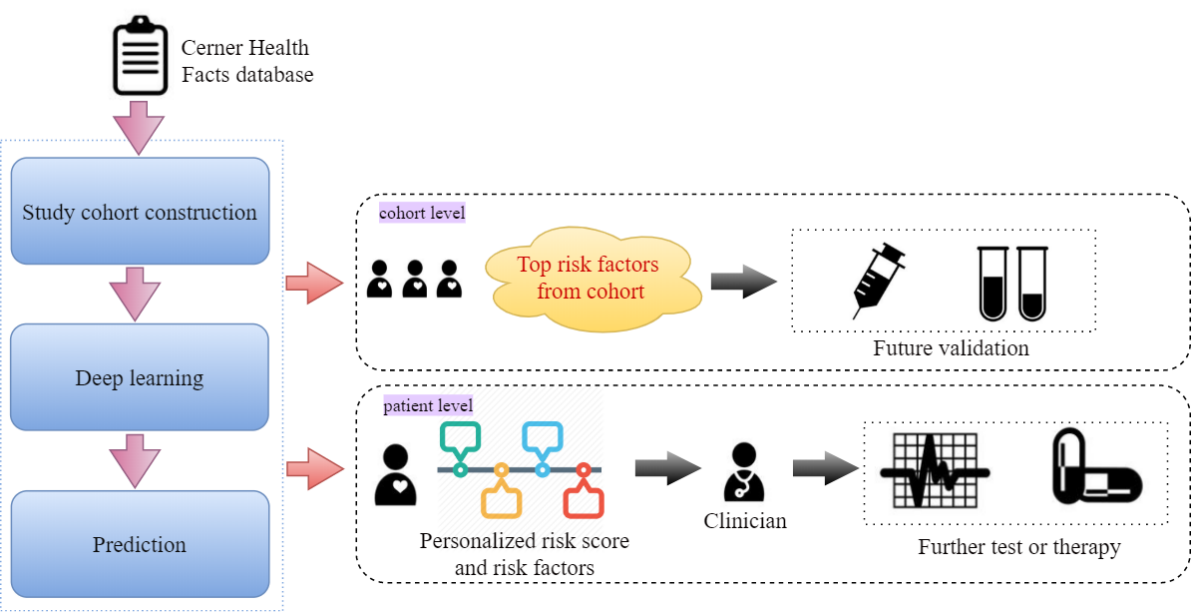
The workflow of the prediction and risk analysis of asthma exacerbation.

The TSANN model was trained to evaluate the risk of asthma exacerbation given the observed time window. The main outcomes of the proposed method are (1) a score that measures the risk of asthma exacerbation for each patient and (2) visualization of the results, including a personalized heatmap identifying the importance of each clinical variable in the observed time window, cohort-level risk factors, and their temporal distributions among patients. On the basis of the outcomes, further data mining or clinical trials can be carried out for validation. For example, cohort-level factors will help data scientists reduce labor and expertise in collecting candidate risk factors from the literature before conducting a regression analysis. Patient-level factors will facilitate physicians and patients in better understanding disease progression. The workflow of this research is shown in Figure 1.

**Table 1:** Defined terms for asthma exacerbation prediction.

Term	Definition
Index date	The date of the first diagnosis of asthma in a patient’s EHR^a^
Exacerbation date	The date of the first diagnosis of asthma exacerbation after the index date
Case group	Patients with asthma and later asthma exacerbations within 365 days and satisfying the inclusion and exclusion criteria
Control group	Patients with asthma but without exacerbations within 365 days and satisfying the inclusion and exclusion criteria
Prediction date	Training set: for the case group, the visit date before the exacerbation date; for the control group, the penultimate visit date within 365 days: Testing set A: the fifth visit starting from the index date Testing set B: defined analogously to the training set
Observed time window	The time window between the index date and the prediction date

^a^EHR: electronic health record.

### Selection of Study Subjects

The subjects in the study were patients with a diagnosis of asthma. The inclusion and exclusion criteria derived from previous studies [4,56] were as follows.

#### Inclusion Criteria

The subjects in the study were patients with at least one record of asthma diagnosis. The definitions of asthma and exacerbation are as follows.

##### Asthma

1. Asthma diagnosis codes were provided according to the International Classification of Disease Code (ICD; ICD-9 code 493.xx or ICD-10 code J45.xx). This is the first occurrence of asthma in the patient’s EHR.
2. At least one of the asthma medications was prescribed on the asthma diagnosis date (the index date). Asthma medications include short-acting beta agonists, inhaled corticosteroids (ICS), long-acting beta agonists (LABA), leukotriene receptor antagonists, anticholinergics, and ICS/LABA combinations.

##### Asthma Exacerbation

1. Asthma (ICD-9 code 493.xx or ICD-10 code J45.xx) was given as a primary diagnosis for an ED visit or hospitalization.
2. At least one oral corticosteroid treatment was received.

#### Exclusion Criteria

To allow the data to better fit for machine learning models, we excluded the following patients:

1. Those with missing or unclear time information (eg, with a wrongly recorded format of time stamps)
2. Those with a gender other than male or female
3. Those whose number of visits is <5 in the observed time window

**Figure 2:**
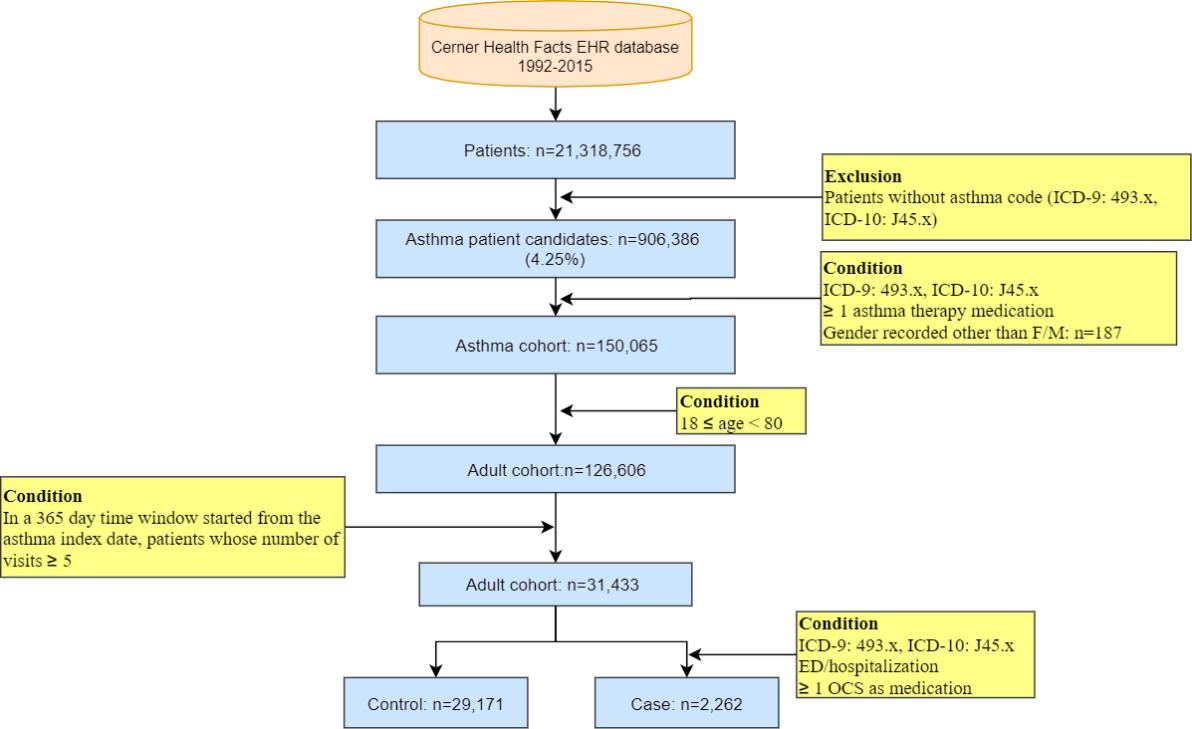
Cohort selection process for the study of asthma exacerbation. ED: emergency department; EHR: electronic health record; ICD: International Classification of Disease Code; OCS: oral corticoids.

This study only focused on adult patients aged between 18 and 80 years. In the end, 31,433 individuals remained, including 2262 cases and 29,171 controls (case by control ratio approximately 1:13). The cohort selection process is shown in Figure 2. A detailed descriptive analysis of the cohort is shown in Multimedia Appendix 1.

### Time-Sensitive Attention Neural Network

#### Model Overview

**Figure 3:**
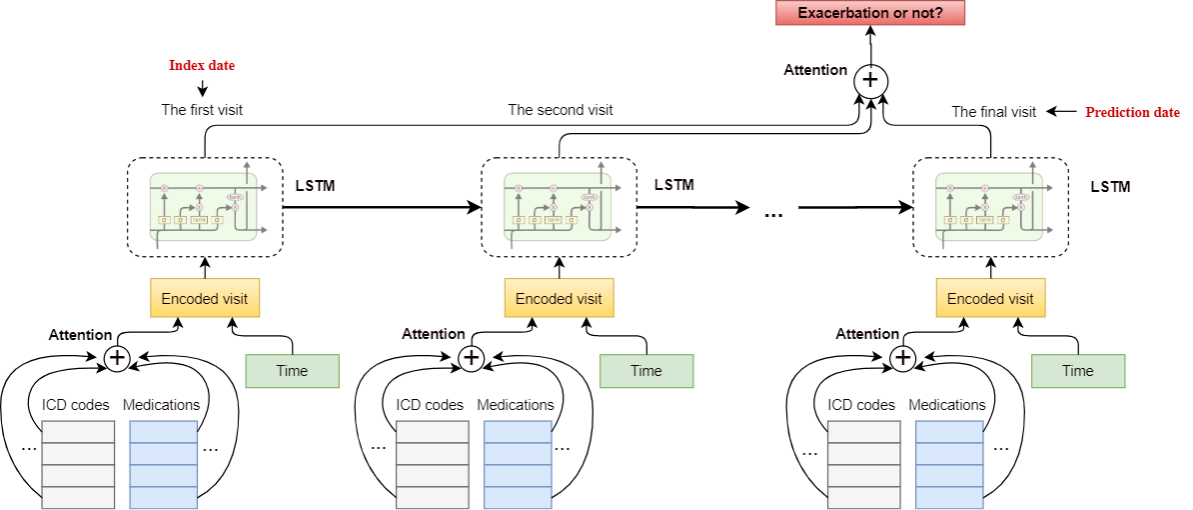
Overview of the time-sensitive attentive neural network model for asthma exacerbation prediction. ICD: International Classification of Disease Code; LSTM: long short-term memory.

TSANN takes the whole sequence of clinical variables in the observed time window as inputs and outputs the probability of asthma exacerbation (Figure 3). The architecture of TSANN is based on LSTM and strengthened by the addition of hierarchical attention and elapsed time embeddings.

For each visit, multiple clinical variables were encoded in the input layer and averaged through the code-level attention mechanism. The elapsed time embedding is attached to each visit as complementary information to indicate the time interval between the date of each visit and the prediction date. LSTM then accepts the sequence of encoded visits as inputs and outputs further encodings for each visit. The visit-level attention layer is then applied to the outputs of the LSTM to summarize all the visits for each patient. Finally, by feeding the output of visit-level attention into the Softmax function, a probability indicating the risk of disease onset is generated.

#### Input

The inputs of the model consist of 2 types of features. One type is clinical concepts (we use *clinical concepts* and *clinical variables* interchangeably), including ICD codes, medications, and demographic features. All ICD-10 codes were converted into ICD-9 based on predefined mappings [57] because very few diagnosis codes in our data set were encoded by ICD-10 as the data collection time range is between 1992 and 2015, but the implementation of ICD-10 started in October 2015. All medications were normalized to their generic names. The demographic features included age, gender, and race, which were only taken as inputs on the prediction date. Using a projection matrix 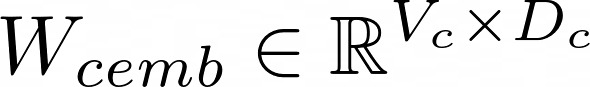
(*V*_c_: concept vocabulary size and *D*_c_: concept embedding dimension), we mapped each clinical concept into a concept-embedding vector:


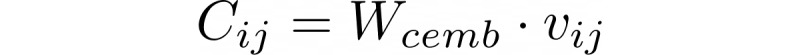


where *C_ij_* is the generated concept-embedding vector and 
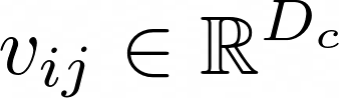
 is the one hot vector denoting the existence of clinical concept *j* in visit *i*.

The other feature type is time features, which indicates the occurrence time for each clinical variable. Intuitively, variables with different time stamps would behave differently in prediction. For instance, in many cases, a clinical event that happened several days ago would play a more important role than one that happened several months ago. Meanwhile, due to the nature of data irregularity and deficiency of EHRs, successive visits always have diverse time intervals [23], which makes it indispensable to consider the time elapsed when conducting predictive modeling.

Elapsed time embeddings were introduced to represent the relative time gap for each clinical concept. Specifically, taking the time of the prediction date *T*_0_ as a pivot, the time attribute of each clinical concept is the absolute difference between its occurrence time *T*_i_ and *T*_0_, that is, the relative time gap *T*_0_ -*T*_i_. As the observed time window has an upper bound of 365 days, the vocabulary size *V*_t_ of the time embeddings was set as 365. We applied a matrix 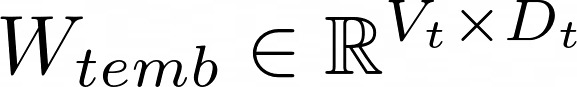 to project each time value to an *m*-dimension vector. Unlike the clinical concept embeddings, elapsed time embeddings are fed into the model after the code-level attention and assigned to each visit. The equation to obtain the elapsed time embedding for each visit is analogous to that for concept embeddings, where: 
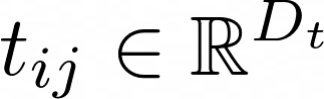



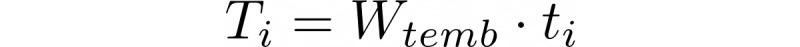


Specifically, the minimum time unit in this study was set as *day*.

#### Code-Level Attention

Attention is a mechanism specifically designed for deep neural networks that acts as an information filter; meanwhile, it can alleviate information loss when dealing with long sequences. It selects important sequence steps by assigning them different weights [58,59]. Through attention, each clinical concept is assigned a weight such that important concepts would have larger weights than the others. We adopted the attention mechanism from Yang et al [60], in which the weight of each variable is generated according to the sequence and context vector. Specifically, given the set of codes 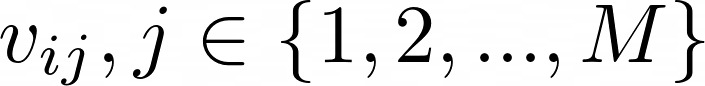 in the *i*th visit, the encoded representation for visit *v*_i_ can be generated by:


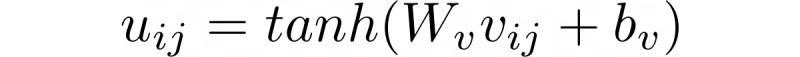



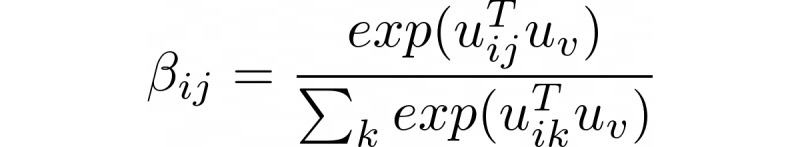



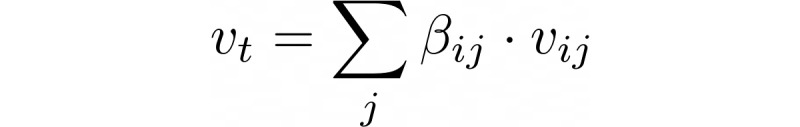


where *W*_v_ and *b*_v_ are the weight and bias for matrix transformation, *u*_ij_ is the attention vector for each code *j* in *v*_i_, *u*_v_ is the context vector for *v*_i_, which is randomly initialized and updated during training, and *_ij_* is the attention weight for the concept *Vij* based on which we can generate its final weight. By assigning time embeddings to the *i*th visit *v*_i_, the representation of each visit is updated as 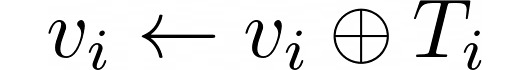, where 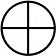 denotes the matrix concatenation.

#### Visit-Level Attentive LSTM Layer

Taking the encoded representation of each visit as input, LSTM models the sequential information in the observed time window and obtains the summarization at the final step (the prediction date). The advantage of LSTMs over traditional RNNs is that they can alleviate the gradient vanishing problem and are thus able to retain longer *memories* from prior time stamps [61,62]. LSTMs are implemented by several matrix multiplications and nonlinear transformations that aim to mimic the memory mechanism of human brains, which are called *gates*, signifying that the network can select effective information and abandon useless information. The equations of the LSTMs are as follows:


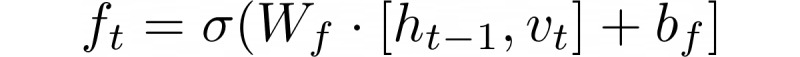



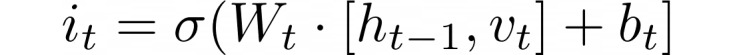



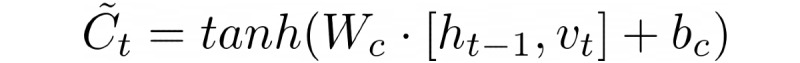



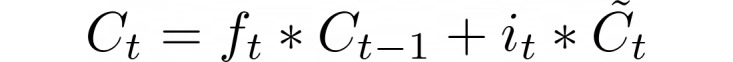



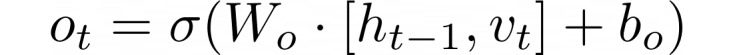



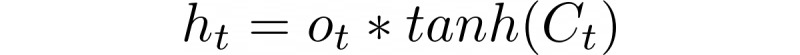


where *W*s and *b*s are the weights and biases for different gates or cells (*f*_t_: forget gate, *i*_t_: input gate, *C*_t_: memory cell, *o*_t_: output gate, and *h*_t_: hidden cell) and *σ* is the activation function, such as *Tanh* or *sigmoid*.

By assigning attention weights to the outputs of LSTM from each step, we can weight each visit in the observed time window and obtain a summary of these visits as *r*_p_:


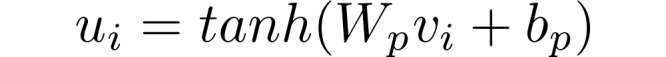



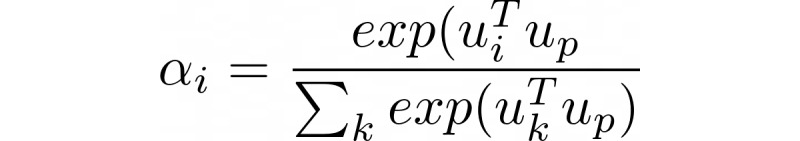



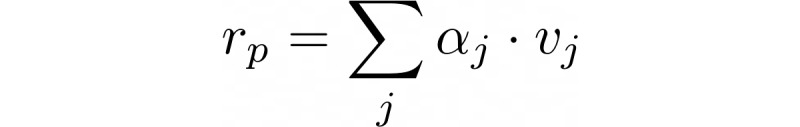


where *W*_p_ and *b*_p_ are the weight and bias for matrix transformation, *u*_i_ is the attention vector for each visit *i* given *v*_i_, *u*_p_ is the context vector, and _j_ is the attention weight for each visit *v*_j_. This process can be seen as a simulation of the diagnosis procedure of a clinic visit, during which a physician would look back into a patient’s EHR, measure the impacts of each historical clinical event, and make the final decision.

#### Output

The visit-level attention layer compresses all the information in the observed time window into a fixed-length vector *r*_p_. The output of attention goes through a fully connected layer with nonlinear activation. Finally, a Softmax function is applied to generate the prediction probability, *P:*


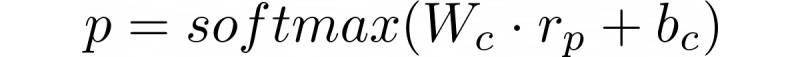


where *P* is used as the score to evaluate the risk of developing asthma exacerbation.

### Evaluation

Area under the receiver operating curve (AUC) is widely used as an evaluation metric for predictive models, which reflects a balance between sensitivity and specificity [63]. According to the prediction probability *P* (between 0 and 1) for each instance, the AUC value is generated by setting different cutoffs. The methods listed in Table 2 were compared in our experiments.

**Table 2:** The methods used for comparisons.

Method	Note
LR^a^	A popular conventional machine learning algorithm [64], usually serving as a strong baseline in predictive modeling [27]. The input of LR is a fixed-length feature vector that denotes the frequencies of each variable. For LR considering time, we associate each variable with its time stamp and expand the vocabulary. We did not use *day* as the time unit as it would have introduced a greater number of variables (ie, 12,390×365 [the code vocabulary size×the maximum number of days]), which would have been too sparse and difficult for computation. Instead, we set month as the time unit, and finally, 148,680 distinct clinical variables were generated. We employed the Synthetic Minority Oversampling Technique [65] to help alleviate the problem of data imbalance
MLP^b^	The MLP model used in this study contains 1 input layer and 1 Softmax layer [66]. The representations of all the codes were averaged on each dimension after being projected to the embedding space for each patient
LSTM^c^	The basic LSTM algorithm, taking the sequence of the clinical variables as input ordered by time. The variables in each visit are averaged
ALSTM^d^	Comprising 1 layer of LSTM and 1 layer of attention
TLSTM^e^ [23]	The time-aware LSTM model, which is one of the state-of-the-art predictive models. In TLSTM, the time gap is used to compute the information decay in the LSTM unit
RETAIN^f^ [27]	A two-layer attention model, which is another state-of-the-art model for the prediction of disease onset. In RETAIN, the time features are not embedded as vectors but real values denoting the gaps from the first visit
ATTAIN^g^ [32]	A modification of TLSTM with special types of attention mechanisms added (flexible attention). It also uses a similar time decay function as RETAIN. We implemented it ourselves using TensorFlow
TSANN^h^-I	The proposed TSANN model but with the second attention layer removed. Prediction is based on the final state of LSTM
TSANN-I-step	Apply the time-encoding method from Song et al [39] on TSANN-I. In TSANN-I-step, although time was also encoded using a vector, it only showed the order of each visit, for example, 1, 2, 3 for consecutive visits, but not the actual elapsed time
TSANN-II	A complete version of the proposed TSANN model

^a^LR: logistic regression.

^b^MLP: multilayer perceptron.

^e^LSTM: long short-term memory.

^d^ALSTM: attention long short-term memory.

^e^TLSTM: time-aware long short-term memory.

^f^RETAIN: Reverse Time Attention model.

^g^ATTAIN: Attention-based Time-Aware Disease Progression.

^h^TSANN: time-sensitive attentive neural network.

For evaluation, we first split the data into a training set and a held-out testing set with a ratio of 8:2. Furthermore, five-fold cross-validation was performed on the training data set for parameter tuning. During cross-validation, a grid search was applied to tune the hyperparameters including learning rate (0.0005, 0.001, 0.005, 0.01), l2 penalty (0.0001, 0.0005, 0.001), batch size (32, 64, 128), activation function for LSTM (ReLU [40] and Leaky_ReLU [41]), whether to add batch normalization [45], and the optimizer selection between RMSprop [42] and Adam [43]. We then averaged the AUCs of each epoch (up to 30 epochs) across five folds to obtain the best training epoch. The optimal hyperparameters were adopted to retrain the model on the entire training set and produce the AUC on the testing set. Finally, the hyperparameters for the model TSANN-I, which has the best AUC value, were as follows: batch size=32, concept embedding dimension=100, time embedding dimension=20, Adam as the optimizer with learning rate=0.001, l2 penalty=0.0001 for all parameters, Leaky_ReLU as the activation function, and adding batch normalization before Softmax. The codes for RETAIN and TLSTM were reused from the respective studies. All other deep learning models were implemented with TensorFlow [44] and trained on Nvidia Tesla V100, Quadro P6000, and Titan XP GPUs. We shared our code on GitHub to facilitate other researchers [67].

## Results

### AUC Values

AUC values with (+time) and without the consideration of time (–time) on testing set A (the primary evaluation set) are shown in Table 3. In the table, for TLSTM, we only considered a *+time* version as it is defined as a time-aware variant of LSTM, and for multilayer perceptron (MLP), LSTM, attention long short-term memory (ALSTM), TSANN-I, TSANN-I-step, and TSANN-II, we used the elapsed time embeddings introduced in this study to include time.

**Table 3:** Area under the curve (AUC) values by different models (–time: time information was excluded and +time: time information was included).

Method	AUC^a^–time	AUC+time
LR^b^	0.6447	0.6773
MLP^c^	0.6545	0.6753
LSTM^d^	0.6045	0.6567
ALSTM^e^	0.6346	0.6714
TLSTM^f^	—	0.6548
ATTAIN^g^	0.6119	0.6597
RETAIN^h^	0.6455	0.6882
TSANN^i^-I	0.6692	*0.7003* ^j^
TSANN-I-step	0.6463	—
TSANN-II	*0.6827*	0.6855

^a^AUC: area under the receiver operating curve.

^b^LR: logistic regression.

^c^MLP: multilayer perceptron.

^d^LSTM: long short-term memory.

^e^ALSTM: attention long short-term memory.

^f^TLSTM: time-aware long short-term memory.

^g^ATTAIN: Attention-based Time-aware Disease Progression.

^h^RETAIN: Reverse Time Attention model.

^i^TSANN: time-sensitive attentive neural network.

^j^The optimal value for each column is italicized.

When comparing vertically (different rows) and considering time information, we noticed that TSANN-I achieved the optimal AUC value, improving the strongest baseline (RETAIN) by 1.21% (the difference was significant according to the Wilcoxon test with *P*=.03). Among other methods, TSANN-II achieved a performance comparable with that of RETAIN. The conventional machine learning method logistic regression (LR) behaved better than some deep learning methods but was worse than RETAIN, TSANN-I, and TSANN-II. TSANN-I-step, which only used time embeddings to denote the relative position of each visit, did not produce good results. Although TLSTM and ATTAIN performed well on other tasks, they did not obtain satisfactory results on our data. For results without time, TSANN-I and -II performed much better than others, with a maximum improvement of 2.82%.

When comparing the results horizontally (–/+ time), considerable improvements were observed after adding time information on most methods; for example, TSANN-I obtained a 3.11% improvement. Surprisingly, TSANN-II, when integrating time embeddings, did not improve considerably.

Better performances by TSANN models and considerable improvements after adding the time information could also be observed on testing set B (we did not list the results here but showed them in Multimedia Appendix 1 as it is not our primary evaluation set). As expected, the general results on testing set B were better than those on testing set A, as a sample conveys more complete information.

### Patient-Level Risk Factors

**Figure 4:**
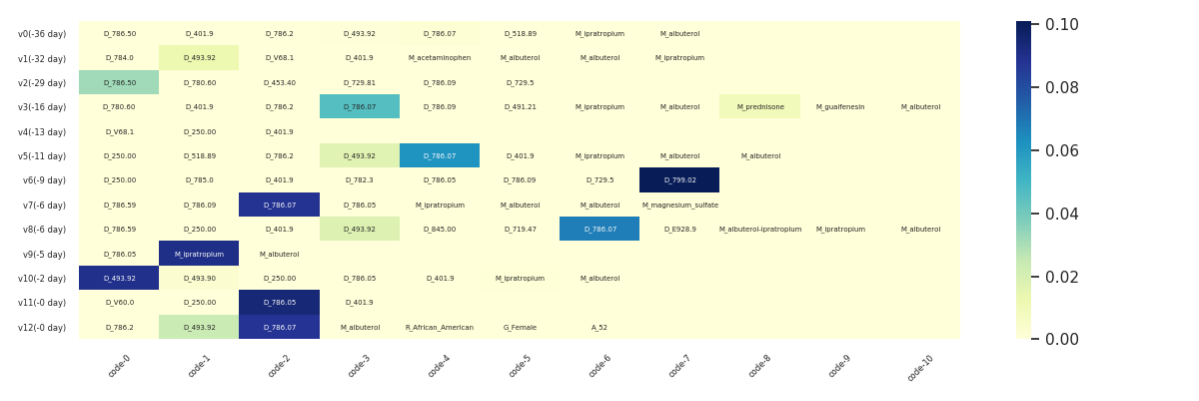
An example of a heatmap with highly associated clinical variables, such as hypoxemia (D_799.02), shortness of breath (D_786.05), and wheezing (D_786.07).

In this study, a heatmap was used for each patient’s EHR for the visualization of highly associated variables or possible risk factors. The heatmap illustrates how each variable behaves in each visit during the progression of asthma. Each grid in the heatmap is colored based on the attention weights derived from the model. The darker an area, the more important the clinical variable and the higher the association it has with exacerbation. For example, Figure 4 shows a case where the symptoms of *hypoxemia*, *shortness of breath*, and *wheezing* (799.02, 786.05, and 786.07 in ICD 9, respectively) were recognized as highly associated variables. A possible explanation might be that the patient’s status of hypoxemia worsened the condition of asthma following symptoms in the breath, and asthma exacerbation was then diagnosed.

These highly associated variables can either be signs of asthma worsening or be triggers for exacerbation, which requires further confirmation by domain experts. Signs including symptoms and treated medications may convey important clues for disease progression and will help clinicians in making final diagnoses, whereas the triggers behaving as personalized risk factors will potentially benefit early interventions. In addition, each heatmap is associated with a probability score derived from equation 15, indicating the risk of the patient in developing an exacerbation (the top row in Figure 4, *predicting 1* indicates *predicting exacerbation*).

### Cohort-Level Risk Factors

We also discovered common highly associated variables at the cohort level using the normalized multiplication of the visit-level and code-level attention weights. We then asked the physician to help distinguish the type of these variables according to their expertise and literature lookup. Some of these variables can be confirmed as risk factors, for example, poor control of respiratory diseases [4] and gastroesophageal reflux disease [44], although others need further validations, such as chest pain, migraine, and use of some medications. The top-ranked variables derived from the model are shown in Table 4. The details of the method and the explanations of these factors are described in Multimedia Appendix 1.

**Figure 5:**
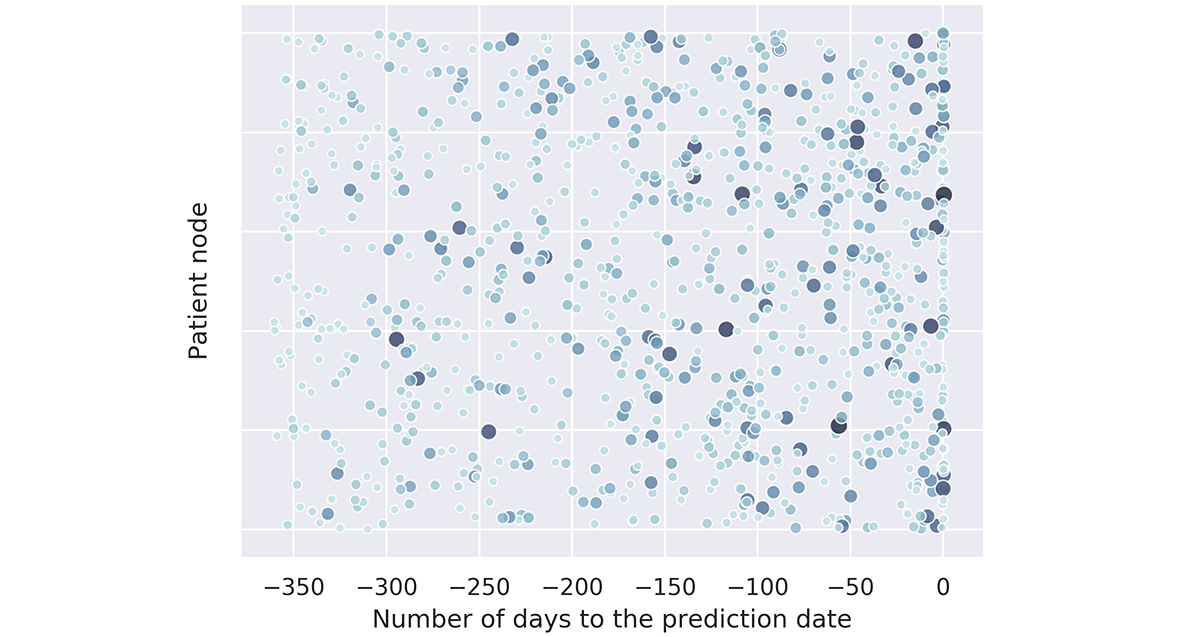
Time distribution of the contribution of the clinical variable gastroesophageal reflux disease is denoted by ICD-9:530.81. ICD: International Classification of Disease Code.

**Figure 6:**
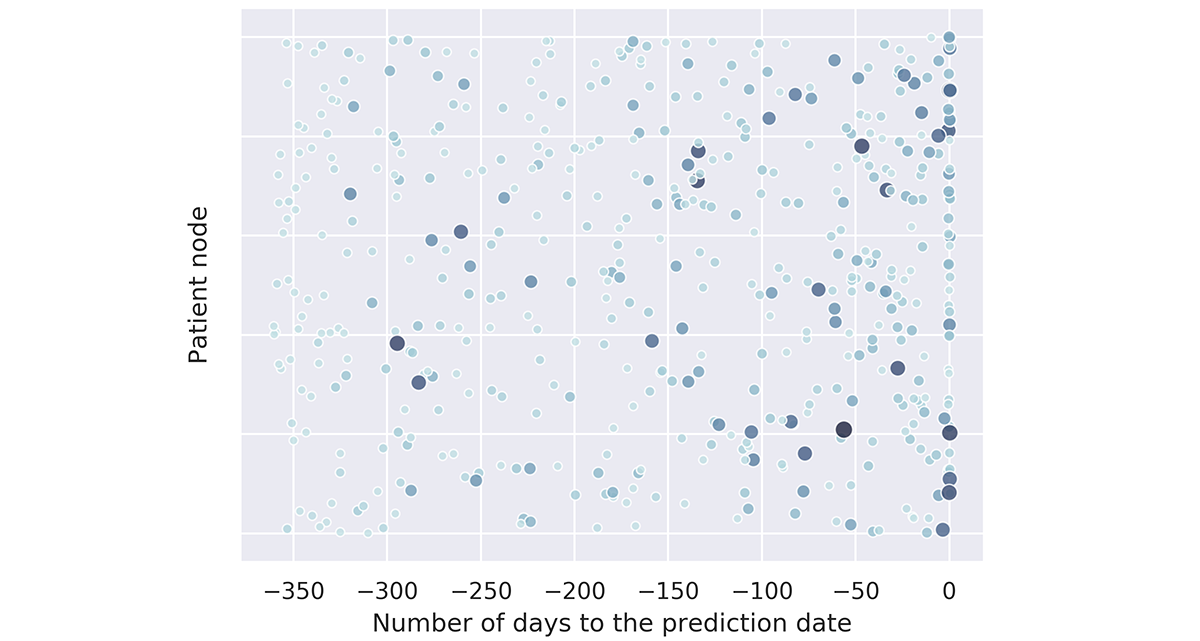
The time distribution of the contribution of the clinical variable fentanyl.

Apart from demonstrating the list of cohort-level factors, using the weights generated by equation 2 and equation 3 in Multimedia Appendix 1, we can also visualize how each clinical variable contributes across time; for example, a variable may behave distinctly among individuals with different action times or different incidences. Figures 5 and 6 present 2 examples in which the time distributions for the clinical variables are displayed through scatters. In these scatters, each circle represents a patient where its size and color depth denote the importance of the corresponding variable. In the figures, the x-axis represents the time gap between the occurrence date of the variable and the prediction date, whereas the y-axis is employed merely for cosmesis. We randomly selected a maximum of 2000 patients to plot this figure.

Figures 5 and 6 were derived from an ICD code (530.81) and a medication (fentanyl), respectively. We observed different effective time ranges for these 2 factors, where the first factor tends to distribute more intensively between the previous 250 to 50 days, whereas the second factor focuses more intensively on the previous 100 days. We hope that these visualizations can help determine the temporal distributions of highly associated factors to aid asthma control.

**Table 4:** Clinical variables with the top-ranked weights (/N stands for the clinical variable presented in N months before the prediction date).

Sr. No.	ICD^a^-9/occurrence time	Medication/occurrence time
1	493.9×asthma/0-5^b^ (meaning diagnosed with asthma multiple times before exacerbation)	Methylprednisolone/0, 1^c^
2	786.07 wheezing/0-2^d^	Prednisone/0, 1, 2^c^
3	496.0 chronic airway obstruction not elsewhere classified/0, 1^e^	Ipratropium/0, 1, 2^c^
4	530.81 esophageal reflux/0^b^	Midazolam/0, 1, 2^d^
5	V46.2 dependence on supplemental oxygen/0^d^	Hydromorphone/0-2^e^
6	787.02 nausea alone/0^d^	Heparin/0, 1^d^
7	786.50 unspecified chest pain/0^d^	Acetaminophen-oxycodone/0^b^
8	V08 HIV infection status/0^e^	Fentanyl/0^e^
9	786.59 other chest pain/0^d^	Methylprednisolone/2-4^e^
10	786.05 shortness of breath/0^d^	Glycopyrrolate/0^b^
11	V58.69 long-term (current) use of other medications/0^e^	Lidocaine/0^d^
12	784.0 headache/0^e^	Dexamethasone/0^d^
13	346.90 migraine, unspecified, without mention of intractable migraine without mention of status migrainosus/0^e^	Promethazine/0^d^
14	V58.66 long-term (current) use of aspirin/0^b^	Atorvastatin/0^d^
15	491.21 obstructive chronic bronchitis with (acute) exacerbation/0^e^	Furosemide/0^c^

^a^ICD: International Classification of Disease Code.

^b^Identified possible risk factors of asthma exacerbations by the domain expert. The authors regard these as containing valuable information.

^c^These medications can be used to treat asthma or control the symptoms of asthma. In this study, it was difficult to determine whether these medications are risk factors as we were unable to investigate the dosage of these medications in the current study. Inappropriate medication use, short-acting beta agonists/inhaled corticosteroids, could also lead to asthma exacerbations.

^d^These factors were symptoms, comorbidities, or combined medications. We believe they were not risk factors for asthma exacerbations.

^e^It could hardly be determined whether these factors caused asthma exacerbations, but they demonstrated high associations. The authors regard these as containing valuable information.

## Discussion

### Principal Findings

Our proposed method obtains the optimal AUC value on the prediction task, with hierarchical attention and elapsed time embeddings as its booster. The visualization also provides useful tracks for a better understanding of disease progression. The primary outcomes of this study are as follows: (1) a new state-of-the-art predictive model for asthma exacerbation prediction was proposed and validated and (2) a reasonable pipeline of disease risk prediction and factor analysis was introduced. Some of the identified risk factors can be validated from the literature, which shows the effectiveness of the method, whereas some other factors, although supportive shreds of evidence were seldom reported in previous studies, offer meaningful insights for further research. The discussions in this section are primarily based on the results of testing set A without additional comments.

### Model Performance

TSANN-I and TSANN-II have the capacity to capture nonlinearities and learn more complex dependency relationships between variables, benefiting from the structure of hierarchical attention and the addition of elapsed time embeddings. It is difficult for LR and MLP to learn temporal dependencies between variables according to their natural structures, which makes them perform worse than TSANNs. However, they obtained better AUCs than LSTM and ALSTM on testing set A, partly because the truncation of EHRs by 5 visits weakens their advantages in modeling longer sequences. Although TLSTM and ATTAIN also integrated the time information, they did not obtain satisfactory results as other methods. It is likely that the combination of the single-layer deep learning structure and the numerical time decay function is insufficient in dealing with more complex temporal patterns on this data set and even confuses the classifiers. RETAIN also has a hierarchical attention structure, as introduced in the Introduction section. However, one of its attention mechanisms is applied to each code-embedding dimension, which is different from ours and requires an additional inference step for interpretation. In addition, our addition of time embeddings enhanced the flexibility of modeling the time information, which contributed considerably to the performance.

A typical characteristic of the EHR data is irregularity, which means that the time gaps between clinic visits are irregular and the visits are often sparsely distributed along the timeline and sometimes are even missing. Thus, the predictive model is responsible for serializing the visits for each patient with consideration of time elapses between continuous visits and reduces the effect of missing data. The comparisons between results with and without time information in Table 3 demonstrate the effectiveness of considering time elapses in this cohort. It might be inferred that the prediction of asthma exacerbation is quite time sensitive and most of the critical risk factors should have been time stamped. For instance, even for a visit just before the prediction date, if its occurrence is several months earlier, its impact would be reduced. Similar cases can also be found in Baytas et al [23], who reported an improvement of 6% from LSTM to TLSTM. In comparison, for TSANN-I-step, although time embeddings were also used, they were only used to denote the relative position of each visit in the sequence but lacked the ability to represent time decays, which can hardly obtain satisfying results here. However, adding time to TSANN-II did not improve much as in other methods, a possible reason might be that the addition of visit-level attention weakens the contribution of time embeddings.

### Risk Factors

As mentioned earlier, the factors identified by this method can be roughly divided into possible risk factors and highly associated factors, for which some pieces of evidence can be found in the literature. Besides, there were still several candidate factors proposed by our model that were seldom reported, for example, HIV infection, or we could not confirm their associations, for example, abdominal pain. One possible reason is that we only considered structured data but not textual information (ie, clinical notes); therefore, that disease or symptom may not be detailed enough to understand given only a code (ie, we know abdominal pain but do not know in which part). Furthermore, according to the AUC values of the model, the results may not be precise enough and still need to be improved. Overall, our method is completely data driven, without any predefined candidate risk factors by experts, which is different from most studies based on regression analysis [7]. We expect that our method can provide compensational information and some new findings can be further validated by clinicians or researchers.

### Error Analysis

We analyzed patient samples that are likely to be false-positives or false-negatives according to the prediction probability (as we did not require an output label but only a probability indicating the risk of each patient). One possible reason for the likely false-negatives (ground truth is case, but the predicted probability for case is quite low) is the data missing problem. For example, patient A had some respiratory symptoms such as asthma, shortness of breath, and chronic airway obstruction about 6 months before the prediction date; however, all the diagnosis codes were related to heart disease and hypertension. Therefore, it is likely that some symptoms that might serve as better indications were missing. On the other hand, one explanation for some false-positives (ground truth is control, but the predicted probability for control is quite low) is the difficulty in evaluating the severity of certain diseases or symptoms. For example, patient B had continuous respiratory symptoms such as chronic airway obstruction, but without any laboratory test values or knowledge of the drugs, it is difficult to determine whether these symptoms worsened or were already well controlled. To mitigate these factors, it is desirable to integrate more variables and background knowledge into the model in the future.

### Comparison With Prior Work

One of the advantages of machine learning over statistical analysis is that it can make predictions on unseen samples [15], and it might be much easier to be deployed in real-world applications. Although many studies have focused on asthma exacerbation prediction, the majority of them belong to statistics, as they did not test their model on held-out data sets [68,69]. Among other machine learning studies, multiple conventional models have been explored, such as classification and regression tree [11,70], random forest [71], LR [72], and support vector machines [16]. However, none of these previous studies used deep learning as we know. Compared with these conventional machine learning methods, our deep learning–based method has multiple advantages. First, no feature engineering is needed, which will extremely reduce the laborious cost and expertise at the first step, for example, in comparison, Luo et al [20] included 235 features designed by multiple clinical experts as inputs that might cost a lot, but we input all the clinical codes to the model and kept their original formats without any feature selection. Second, LSTM structures can integrate any temporal patterns; thus, dependencies between variables can be easily modeled. Third, deep learning methods usually obtain better performances compared with conventional machine learning methods because of their capacities in modeling complex data structures [22,23,27], which was also proved in our experiments (compared with LR).

Furthermore, compared with previous studies, we are the first to make comprehensive visualization and personalization over the associated factors. One paper mentioned *personalize*, but it only discussed it as a future possibility [16]. In comparison, our method showed not only cohort-level factors but also temporal-based personalized risks and factors, which would greatly facilitate precise medicine. Meanwhile, as we did not limit our input to the predefined factors, we were able to find new potential risk factors. However, one drawback of deep learning–based methods compared with the previous shallow methods is the lack of interpretability from some perspectives, for example, they can hardly report statistical evaluation measures such as *P* values and CIs, which might need further exploration.

### Limitations and Future Work

Using deep learning, we offered a novel means of identifying possible risk factors and predicting the risk of asthma exacerbation. However, this study has some limitations. First, for the model interpretation part, how multiple clinical variables interact with each other needs further exploration; simply considering each variable independently but ignoring the dependency patterns between them might be insufficient for interpretation, for example, the prescription of a drug might be closely associated with a disease or symptom. Second, structured EHRs have their own drawbacks, such as data irregularity, sparsity, and noise. Thus, some potential risk factors for asthma exacerbations might not be recorded or might even be incorrectly recorded in EHRs. As a result, information integrity cannot be guaranteed. We may need to find ways to make the data complete and more reliable, such as including information from textual reports or patient surveys. Third, it is still difficult for computer programs alone to distinguish between asthma symptoms and risk factors, and knowledge injection is needed in the future. Finally, the performance of the model still has room for improvement. It might be boosted further by designing more powerful structures or including background knowledge.

### Conclusions

In this paper, we proposed an attentive deep learning–based model for asthma exacerbation prediction and employed elapsed time embeddings to model the time decays. By leveraging the weights of the model, we not only generated personalized heatmaps and specific risk scores at the individual level but also identified possible risk factors at the cohort level. Compared with previous studies, our model is effective in modeling time information and obtains better overall AUCs. As the model is completely data driven and relies little on feature engineering, it can easily be generalized to other prediction tasks. To the best of our knowledge, this is the first study to predict asthma exacerbation risks using a deep learning model that includes elapsed time embeddings. Some of the top-ranked risk factors identified have gained supporting evidence from previous medical studies, which proved that our method has good reliability and accuracy.

## Appendix

Multimedia Appendix 1 Supplementary materials.

## Abbreviations

ALSTM: attention long short-term memory
ATTAIN: Attention-based Time-aware Disease Progression
AUC: area under the receiver operating curve
EHR: electronic health record
ICD: International Classification of Disease Code
ICS: inhaled corticosteroids
LR: logistic regression
LSTM: long short-term memory
RETAIN: Reverse Time Attention model
RNN: recurrent neural network
SABA: short-acting beta agonist
TLSTM: time-aware long short-term memory
TSANN: time-sensitive attentive neural network
