# Supplementary material for: Asthma Exacerbation Prediction and Risk Factor Analysis Based on a Time-Sensitive, Attentive Neural Network: Retrospective Cohort Study
Source: J Med Internet Res. 2020 Jul 31;22(7):e16981. doi: 10.2196/16981 (PMC7428917; doi:10.2196/16981)
Supplement: Multimedia Appendix 1 [file jmir_v22i7e16981_app1.docx]

# Multimedia Appendix

This is a Multimedia Appendix to a full manuscript published in the J Med Internet Res. For full copyright and citation information see http://dx.doi.org/10.2196/jmir.xxxx

### Inclusion/exclusion criteria

#### Inclusion criteria

The subjects in the study were patients with a diagnosis of asthma. Cases are patients with the diagnosis of asthma and asthma exacerbation in 365 days awhile controls are patients without exacerbations. The definitions of asthma and exacerbation are:

*Asthma*

a) An asthma diagnosis code was given according to the International Classification of Disease Code (ICD) (ICD-9 code 493.xx or ICD-10 code J45.xx). It is the first occurrence of asthma in the patient’s EHR.

b) At least one of the asthma medications was prescribed on the asthma diagnosed date (the index date). The asthma medications considered include Short-Acting Beta Agonists (SABA), Inhaled Corticosteroids (ICS), Long-Acting Beta-Agonists (LABA), Leukotriene Receptor Antagonist (LTRA), anticholinergics, and ICS/LABA combinations.

*Asthma exacerbation*

a) The asthma diagnosis was given as a primary diagnosis code according to the ICD code (ICD-9 code 493.xx or ICD-10 code J45.xx) for an Emergency Department (ED) visit or hospitalization

b) At least one Oral Corticosteroid (OCS) treatment was received.

#### Exclusion criteria

To let the data better fit for machine learning, especially RNN-based models, we excluded the following patients:

(1) with unclear time information (i.e. with wrong timestamps due to data conversion errors);

(2) of gender other than male or female;

(3) whose number of visits are less than 5 in the observed time window according to the average number of visits (5.78) between the index and the exacerbation date.

### Descriptive analysis on the cohort

In this study, we narrow our focus to the visits within a period 365 days after the index date. Table 1 is a general descriptive analysis for demographic characteristics of the cohort, and Table 2 shows the statistical numbers of the experiment cohort. For gender, we did not recognize significant differences (P>0.05) in distributions between case and control. The distribution of ethnicity in our database was imbalanced, and there were significant differences according to the p-value (P<0.001). However, our deep learning methods remained agnostic to these imbalanced distributions, and we just let the model learn the weights automatically.

Table 1. Descriptive analysis for demographic characteristics of the cohort. Chi-square test is used for categorical variables. The values in the brackets are percentage in the corresponding group.

| **Category** | **Value** | **Case (%)** | **Control (%)** | **P-value** |
| --- | --- | --- | --- | --- |
| Gender | Female | 1,648 (74.18) | 21,335 (73.14) | 0.473 |
|  | Male | 5,84 (25.82) | 7,836 (26.86) |  |
| Race | African American | 752 (33.24) | 6,605 (22.64) | < 0.001 |
|  | Caucasian | 1,270 (56.15) | 20,148 (69.07) |  |
|  | Biracial | 5 (0.22) | 41 (0.10) |  |
|  | Asian | 5 (0.22) | 137 (0.47) |  |
|  | Native American | 17 (0.75) | 196 (0.67) |  |
|  | Hispanic | 46 (2.03) | 447 (1.53) |  |
|  | Pacific Islander | 0 (0) | 21 (0.07) |  |
|  | Asian/Pacific Islander | 0 (0) | 4 (0.01) |  |
|  | Other | 167 (7.38) | 1,583 (5.42) |  |

Table 2. Statistics on numbers of the experiment cohort.

| **Type** | **Item** | **Value** |
| --- | --- | --- |
| visit | Max number of visits per patient | 174 |
|  | Min number of visits per patient | 5 |
|  | Average number of visits per patient | 10.24 |
| code* | Max number of codes per visit | 184 |
|  | Min number of codes per visit | 1 |
|  | Average number of codes per visit | 8.72 |

^*^All diagnosis code, medication, demographic features were taken as codes.

### Prediction Date Selection

For the training set, the previous visit before the exacerbation (for case) or the penultimate visit within the observed time window (365 days) (for control) was selected.

For the testing set, the fifth visit starting from the index date was selected as the prediction date. We did not select a fixed time point as the prediction date since the time gaps between asthma index and exacerbation could be quite diverse (Figure 1), which is different from predictions on the ICU patients mentioned in [1].

We have the following considerations for doing so:

1) Selecting the previous visit before the event (for training) makes a positive sample more distinguishable from negative samples since it includes more complete information for causing the event.

2) Simply selecting the prediction date (for testing) according to the index date in this study may lead to a more imbalanced dataset (i.e. more controls) given the diverse gaps between the index and the event. We select the fifth visit as an estimated time for doing predictions, so as to keep more records for prediction, based on the fact of the average number of visits between index and exacerbation is 5.78 and the number of case patients with over 5 visits is 4,279 out of 4,754 (90%) (if we set the minimum number of visits as 3 to enable the sequential dependency learning), as well as to keep more patients for experiments. Originally, we had 61,105 patients, and after truncating using the minimum number of visits five, we got 31,433 patients at last. We also defined the testing set analogous to how we selected patients in the training set, and showed the results in this Multimedia Appendix as the upper bound of the performance.

Figure 1. The distribution on gap between asthma index and exacerbation. The x-axis is the number of patients (in the original cohort) and the y-axis is the time gap in month.

Figure 2 shows two cases where the prediction date is defined based on different criteria, where the training set and testing set B were defined according to (a), and testing set A to (b). There have been continuous debates in this domain on how to select the prediction date. We think it is can be acceptable to use (b) if one is an inpatient or ICU patient, while it is better to use both (a) (for training) and (b) (for testing) if one is an outpatient. Meanwhile, we can always choose any way to define the training set to maximize our outcome.


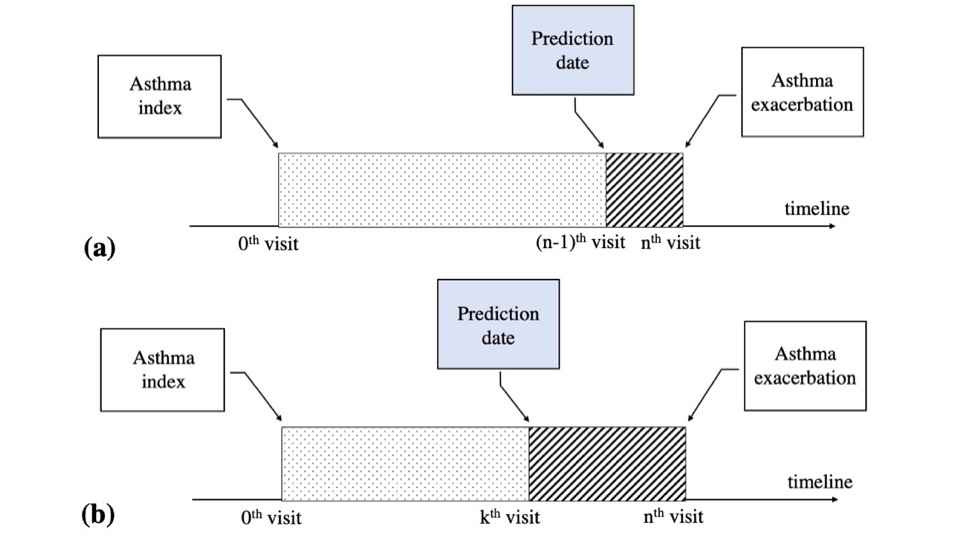


Figure 2. Two cases of defining the prediction date: (a) based on the event (asthma exacerbation); (b) based on the index (asthma index).

### Hyperparameters

During cross validation, grid search was applied to tune the hyperparameters including learning rate [0.0005, 0.001, 0.005, 0.01], l2 penalty [0.0001, 0.0005, 0.001], batch size [32, 64, 128], activation function for LSTM [None, ReLU[2], Leaky_ReLU[3]], whether to add batch normalization[4], and solver RMSprop[5] or Adam[6] so that the model could obtain the best AUC value within each fold. Then we averaged the AUCs of each epoch (up to 30 epochs) across five folds to get the best training epoch. The optimal hyperparameters were adopted to retrain the model on the whole training set and produce the AUC on the testing set. For the deep learning models, we set the dimension of code embeddings as 100 and time embeddings as 20.

Finally, the regularizers for the deep learning models were set as: none for LR-dense (with and without time), L2 with λ=0.0001 for LSTM, ALSTM, TSANN-I and TSANN-II (with and without time), and L2 with λ=0.001 for RETAIN (with and without time) and TLSTM. The solver for optimizing the deep neural network were RMSprop for the LSTM-based models and Adam for LR-dense. LR-sparse was implemented using the Scikit-learn package in Python3, and the parameters were set to be L2 with penalty 1e-4 for the model with time and L2 with λ=0.001 for that without time. Batch size of 32, batch normalization and Leaky-ReLU were used for all the deep learning models. Codes for RETAIN and TLSTM were provided by the respective authors, and all other deep learning models were implemented with TensorFlow[7] and tested on Nvidia Tesla V100, Quadro P6000 and Titan XP GPUs.

### Results on Testing set B

The results for testing set B behave as the upper bound of the models to show their capacities if ideal prediction time was given, which could possibly be approached with the help of some domain knowledge (e.g. an experienced doctor may have the sense of the proper time to make the prediction for a certain patient).

Table 3. AUC values by the proposed models compared with baselines. (+/- stands for the improvement of adding time info).

| **Method** | **Event as the anchor**  **(Testing set B)** | | |
| --- | --- | --- | --- |
|  | **Without**  **time** | **With**  **time** | **+/-(%)** |
| *LR-sparse* | 0.5313 | 0.5906 | +5.93 |
| *LR-dense* | 0.673 | 0.7877 | +11.47 |
| *LSTM* | 0.6160 | 0.778 | +16.2 |
| *ALSTM* | 0.6408 | 0.8044 | +16.36 |
| *TLSTM* | - | 0.7267 | - |
| *RETAIN* | 0.6699 | 0.7761 | +10.62 |
| *TSANN-I* | **0.6843** | **0.8202** | +13.59 |
| *TSANN-II* | 0.6656 | 0.8117 | +14.61 |

*the optimal value for each column is marked in bold.

### Cohort-level Risk Factors

Among the demographic characteristics, gender and race were also identified as risk factors, in which African Americans and Caucasians were recognized as in more risk of asthma exacerbation. We only focus on the discussion of diagnosis codes and medications in the current study.

The cohort-level risk factors are extracted based on the attention weights of the model.

Let the operator “-” denotes *previous*, e.g. -35 days means 35 days prior to the prediction date, to simplify the problem, we firstly converted the granularity of each timestamp from day to month, e.g. -35 days was converted to -2 months and -15 days was converted to -1 month. For each patient, we calculated the weights of each clinical variable based on weights from the two attention layers and accumulated the weights if two clinical variables fall into the same month. The weighted clinical variables were then ranked in a descending order and a weighted ranked variable list was generated. Mathematically, given a matrix of variables (row: each visit, column: variables in each visit), a list of visit-level weights *α*s (weight for each visit), and a matrix of code-level weights *β*s (weight for each variable in each visit), the normalized weight *w*_ij_ for the *j*th variable in the *i*th visit can be computed by:

$w_{ij}=\frac{\exp(\alpha_{i}\beta_{ij})}{\sum_{m} \sum_{n} \exp(\alpha_{m}\beta_{mn})}$ (2)

where m is the number of visits and n is the number of variables in each visit. The relative weight for this variable is further adjusted by the predicting probability *P*:

$\overline{w}_{ij}=p\times w_{ij}$ (3)

Then, we averaged the weight of each variable across the predicted case patients to get the weighted variables. We can also count how many patients are associated with a certain variable. Those variables with bigger weights and frequencies were taken as the cohort-level risk factors.

It is noticed that some known risk factors of asthma exacerbations were identified. Firstly, poor control of respiratory diseases is one essential risk factors of asthma exacerbations[8]. In Table 4 of the paper, there were some respiratory symptoms and diseases, including *wheezing*, *chronic airway obstruction*, *shortness of breath*, *chest pain*, and *obstructive chronic bronchitis with (acute) exacerbation*. Wheezing and shortness of breath are common symptoms of asthma. Chest pain is also a symptom of asthma exacerbation. Poor controlled bronchitis and pneumonia could induce asthma exacerbation. Although asthma and COPD are different diseases, sometimes they could overlap. Asthma-chronic obstructive pulmonary diseases overlap, termed as ACO, is a complex heterogeneous disease without any clear diagnostic. [9] Secondly, asthma is one of the extra-esophageal symptoms of gastroesophageal reflux disease. [10] Asthma and gastroesophageal reflux are widespread and potentially coexisting diseases. [11] In the study, esophageal reflux was extracted and listed as top 15 diagnosis. This disease could induce asthma exacerbation. Based on the result, two of the top three medications were glucocorticoids. It means that most patients in our database were treated with glucocorticoids in asthma control before the onset of asthma exacerbations. To prevent exacerbations, it is necessary to monitor patients’ responses to these medications. Fourthly, headache and migraine were extracted. Some study showed that adult patients with migraine are at a higher future risk of asthma development. [12] However, some study demonstrated that migraine headache might be associated with poor asthma control. [13] Nevertheless, some analgesic drugs were identified as top-ranked weights risk factors (medications), such as Fentany1 and Acetaminophen-oxycodone. Opioids are potent respiratory depressants, and overdose of opioids could lead to death[14]. Fentanyl, as an opioid drug, was reported by a previous study as a trigger in developing asthma exacerbation[15]. Acetaminophen is a widely used medication for the treatment of pain and fever. There is substantial epidemiological evidence in adults and children that acetaminophen use is associated with asthma symptoms[16,17]. Long-term (current) use of aspirin was extracted in our study. Respiratory reactions triggered by oral aspirin in people with asthma are relatively common. [18]

Ipratropium and midazolam were extracted because they were used to control asthma symptoms. They are not risk factors of asthma exacerbations. It is worth to mention that, 9 of the top 15 medications in Table 4 of the paper can be used to treat asthma or control asthma symptoms. These medications have been extracted may be because they are commonly used. In the study, they should not be treated as risk factors of asthma exacerbations.

There were still several candidate factors proposed by our model that were not previously reported. One possible reason is that we only have considered structured data but not the textual information (i.e. clinical notes) so that a disease or symptom may not be detailed enough to understand given only a code (i.e. we know abdominal pain but don’t know in which part). Furthermore, according to the AUC values of the model, the results may not be precise enough and still need to be improved. Overall, we expect some new findings can be further validated by clinicians or researchers.

# References

1 Rajkomar A, Oren E, Chen K, *et al.* Scalable and accurate deep learning for electronic health records. *npj Digit Med* 2018;:1–10. doi:10.1038/s41746-018-0029-1

2 Nair V, Hinton GE. Rectified linear units improve restricted boltzmann machines. *Proc 27th Int Conf Mach Learn* 2010;:807–14. doi:10.1.1.165.6419

3 Xu B, Wang N, Chen T. Empirical Evaluation of Rectified Activations in Convolution Network. 2015.

4 Ioffe S, Szegedy C. Batch Normalization : Accelerating Deep Network Training by Reducing Internal Covariate Shift.

5 Hinton G, Srivastava N, Swersky K. Neural Networks for Machine Learning. https://www.cs.toronto.edu/~tijmen/csc321/slides/lecture_slides_lec6.pdf

6 Kingma DP, Ba J. Adam: a method for stochastic optimization. In: *Iclr*. 2015. doi:http://doi.acm.org.ezproxy.lib.ucf.edu/10.1145/1830483.1830503

7 Mart´ın Abadi, Ashish Agarwal PB et al. TensorFlow: Large-Scale Machine Learning on Heterogeneous Distributed Systems. 2015. doi:10.1093/library/s4-X.3.339

8 GINA. Pocket Guide for Asthma Management. *Pocket Guid asthma Manag Prev* 2018.

9 Ghosh N, Choudhury P, Subramani E, *et al.* Metabolomic signatures of asthma-COPD overlap (ACO) are different from asthma and COPD. *Metabolomics* 2019;**15**:87. doi:10.1007/s11306-019-1552-z

10 Naik RD, Vaezi MF. Extra-esophageal gastroesophageal reflux disease and asthma: Understanding this interplay. *Expert Rev Gastroenterol Hepatol* 2015;**9**:969–82. doi:10.1586/17474124.2015.1042861

11 Paolo SOLIDORO, Filippo PATRUCCO, Sharmila FAGOONEE RP. Asthma and gastroesophageal reflux disease: a multidisciplinary point of view. *Minerva Med* 2017;**108**:350–6.

12 Peng YH, Chen KF, Liao WC, *et al.* Association of migraine with asthma risk: A retrospective population-based cohort study. *Clin Respir J* 2018;**12**:1030–7. doi:10.1111/crj.12623

13 Dirican N, Demirci S, Cakir M. The relationship between migraine headache and asthma features. *Acta Neurol Belg* 2017;**117**:531–6. doi:10.1007/s13760-017-0764-0

14 Savelloni J, Gunter H, Lee KC, *et al.* Risk of respiratory depression with opioids and concomitant gabapentinoids. 2017;:2635–41.

15 Parmar MS. Exacerbation of asthma secondary to fentanyl transdermal patch. *BMJ Case Rep* 2009.

16 Henderson AJ, Shaheen SO. Acetaminophen and asthma. *Paediatr Respir Rev* 2013;**14**:9–16. doi:10.1016/j.prrv.2012.04.004

17 Acetaminophen May Be Linked to Asthma Risk. https://www.webmd.com/asthma/news/20091105/acetaminophen-may-be-linked-to-asthma-risk#1

18 Morales DR, Guthrie B, Lipworth BJ, *et al.* NSAID-exacerbated respiratory disease: A meta-analysis evaluating prevalence, mean provocative dose of aspirin and increased asthma morbidity. *Allergy Eur J Allergy Clin Immunol* 2015;**70**:828–35. doi:10.1111/all.12629
